# Meniscal mineralisation in little spotted cats

**DOI:** 10.1186/1746-6148-9-50

**Published:** 2013-03-18

**Authors:** Sheila C Rahal, Mauricio G Fillipi, Maria J Mamprim, Hugo S Oliveira, Carlos R Teixeira, Rodrigo HF Teixeira, Frederico OB Monteiro

**Affiliations:** 1 Department of Veterinary Surgery and Anesthesiology, School of Veterinary Medicine and Animal Science - Universidade Estadual Paulista (UNESP), Botucatu, SP, Brazil; 2 Department of Radiology and Animal Reproduction, School of Veterinary Medicine and Animal Science - UNESP, Botucatu, SP, Brazil; 3 Quinzinho de Barros Municipal Zoo, Sorocaba, SP, Brazil; 4 Universidade Federal Rural da Amazônia, Instituto de Saúde e Produção Animal, Belém do Pará, Brazil

## Abstract

**Background:**

The aim of this study was to evaluate the stifle joints of little spotted cats in captivity using radiographic and CT studies. The hypothesis was that these animals would have meniscal mineralisation that could be detectable by imaging studies. Twelve intact little spotted cats (*Leopardus tigrinus*), 2 females and 10 males, aged from 1.5 to 11.11 years old and weighing 1.9–3.05 kg were studied. These animals, which were living in the Quinzinho de Barros Municipal Zoo, had no symptoms or known disease processes at the time of the study. The plain radiographs and computed tomography (CT) scans of both stifle joints were performed under general anaesthesia. Sequential transverse images were acquired on a spiral scanner.

**Results:**

No signs of articular disease were observed in any of the animals. Radiographically, the meniscal mineralisation was detected as an oval radiopacity in the cranial compartment on the mediolateral projection, located within the area of the medial meniscus. On craniocaudal projection, the mineralisation was more difficult to visualise. In one of the animals, it was not possible to identify the meniscal mineralisation in either of the stifle joints. Using CT, meniscal mineralisation was best identified in the transverse plane images.

**Conclusions:**

Meniscal mineralisation appears to be a normal anatomic feature in little spotted cats.

## Background

The stifle joint of quadruped mammals has a medial meniscus and a lateral meniscus that are positioned between the femoral condyles and the tibial plateau [1,2]. The menisci have functions that include load transmission and shock absorption, joint stabilization, joint lubrication and proprioception [3]. A sesamoid in the meniscus denominated lunula or meniscal ossicle is frequently observed in rodents such as rats, mice, hamsters, and guinea pigs [4-8]. The guinea pig has, for example, five lunulae positioned in the cranial aspects of the medial and lateral menisci. The craniomedial lunula is always present, but the others lunulae may be missing (caudomedial, caudolateral, craniolateral and craniosagittal) [7]. However, in a study of 4029 boars of the Landrace and Yorkshire breeds, meniscal ossification was detected in the cranial horn of the lateral meniscus as a single or multiple foci, showing a prevalence rate of 2.6% [9]. Furthermore, meniscal mineralisation has been reported in domestic cats [10], and in large non-domestic cats (lions, tigers, leopards, jaguars, cougars, and pumas) [11,12].

Among the small non-domestic cats, the little spotted cats or tiger cats (*Leopardus tigrinus*) are among the smallest wild cats in Central America and South America, and their body size resembles that of the domestic cat [13,14]. Their body weight ranges between 1.5 and 3.5 kg, and the males are slightly larger than the females [13-15]. They are climbers and hunters, feeding primarily on small mammals but also on birds and lizards [14,16]. The age of their sexual maturity is not clear and may occur up to 11 months, and their longevity has been reported to be 20 years [13,15].

The aim of this study was to evaluate stifle joints of little spotted cats in captivity using radiographic and CT studies. The hypothesis was that these animals would have meniscal mineralisation that could be detectable by imaging studies. To the author’s knowledge, the hind limbs have not been studied in this species. Radiographic evaluation of the hip joint, femur, and sesamoid bones was included to detect possible variation in anatomic features, or signs of a degenerative lesion, that may contribute to the presence or absence of the meniscal mineralization.

## Methods

This study followed the guidelines for the care and use of laboratory animals and was approved by the Ethics Committee of School of Veterinary Medicine and Animal Science (Unesp Botucatu) and by the national environmental and wildlife bureau.

Twelve intact little spotted cats (*Leopardus tigrinus*), 2 females and 10 males, aged from 1.5 to 11.11 years old, weighing 1.9–3.05 kg, were studied (Table 1). The animals, which were living in the Quinzinho de Barros Municipal Zoo, had no symptoms or known disease processes at the time of the study. Groups of two or three animals were housed together in an enclosure with a solarium and received maintenance cat food, meat, vitamin and mineral supplements, and water *ad libitum*.

**Table 1:** Little spotted cat signalment, length of meniscal ossicle and HU values of the meniscal ossicle measured on the transverse CT images, and radiographic Norberg angle

**No.**	**Little spotted cat signalment**	**Length of the meniscal ossicle (cm)**		**Hounsfield unit (HU) values**		**Norberg angle**	
		**Transverse**	**Longitudinal**	**Medium value**	**Maximum value**	**Right**	**Left**
1	2.2 kg 7-y-old male	R: -	R: -	R: -	R: -	108º	109º
		L: -	L: -	L: -	L: -		
2	1.9 kg 6-y-old female	R: 0.2	R: 0.3	R: 660	R: 744	102º	102º
		L: 0.2	L: 0.2	L: 280	L: 338		
3	2.4 kg 4.2-y-old male	R: 0.2	R: 0.2	R: 876	R: 1020	108º	112º
		L: 0.2	L: 0.2	L: 864	L: 919		
4	2.35 kg 2.9-y-old male	R: 0.2	R: 0.2	R: 365	R: 378	106º	105º
		L: 0.1	L: 0.1	L: 418	L: 528		
5	2.65 kg 1.5-y-old male	R: 0.1	R: 0.2	R: 739	R: 761	105º	105º
		L: 0.1	L: 0.3	L: 734	L: 794		
6	3.07 kg 1.10-y-old male	R: 0.1	R: 0.2	R: 684	R: 756	103º	105º
		L: -	L: -	L: -	L: -		
7	2.65 kg 2.2-y-old male	R: 0.1	R: 0.1	R: 309	R: 534	111º	108º
		L: 0.1	L: 0.1	L: 629	L: 712		
8	2.5 kg 11.11-y-old male	R: 0.2	R: 0.3	R: 845	R: 1124	100º	101º
		L: 0.1	L: 0.3	L: 838	L: 1075		
9	2.05 kg 3-y-old female	R: 0.1	R: 0.1	R: 1130	R: 1174	112º	105º
		L: 0.2	L: 0.2	L: 769	L: 840		
10	2.9 kg 8-y-old male	R: 0.2	R: 0.3	R: 631	R: 792	103º	110º
		L: 0.2	L: 0.3	L: 709	L: 823		
11	2.5 kg 10-y-old male	R: 0.2	R: 0.2	R: 745	R: 774	105º	106º
		L: 0.2	L: 0.2	L: 814	L: 914		
12	2.55 kg 2-y-old male	R: 0.2	R: 0.2	R: 807	R: 841	109º	104º
		L: 0.2	L: 0.2	L: 732	L: 794		

Abbreviations: y, years; - , not visible on CT.

### Anaesthetic procedure and imaging evaluation

The radiographic and CT exams were performed under general anaesthesia. After IM premedication with acepromazine (0.05 mg/kg), morphine (0.2 mg/kg) and ketamine (10 mg/kg), anaesthesia was induced intravenously with propofol (5 mg/kg) and maintained with isoflurane.

Green-sensitive film and cassettes with screens were used for the radiographic exam. A focus-film distance of 100 cm was used, with an exposure of 45 kV e 3.2 mAs for the mediolateral view of the right and left stifle joints and an exposure of 50 KV e 3.2 mAs for the craniocaudal and ventrodorsal views of the stifle and hip joints, respectively. Radiographs were evaluated for the presence of meniscal mineralisation and sesamoid bones and for evidence of degenerative joint disease in the stifle joints or the hip joints. The Norberg angle was measured for each hip joint. The anatomic features of the femur and stifle joints were also analysed.

A CT examination was performed with the animal positioned in dorsal recumbency on a foam support, with the hind limbs drawn caudally in a maximal extension position secured by adhesive tape. Sequential transverse images of both hind limbs from the distal third of the femur to the proximal tibia were acquired with a spiral scanner (Shimadzu SCT-7800CT, Kyoto, Japan). The scanning parameters were 120 kVp, 100 mA, 1.0-mm-thick slices, a pitch of 1.0, and 1 s/rotation. The images were reconstructed in MPR using eFilm Workstation™ 2.1.2 software. CT scans were used to confirm the presence of meniscal mineralisation and sesamoid bones. The meniscal mineralisation was measured in the maximum transverse and longitudinal lengths (cm), and the medium and maximum Hounsfield unit (HU) values were determined utilising the transverse imaging plane.

## Results

**Figure 1:**
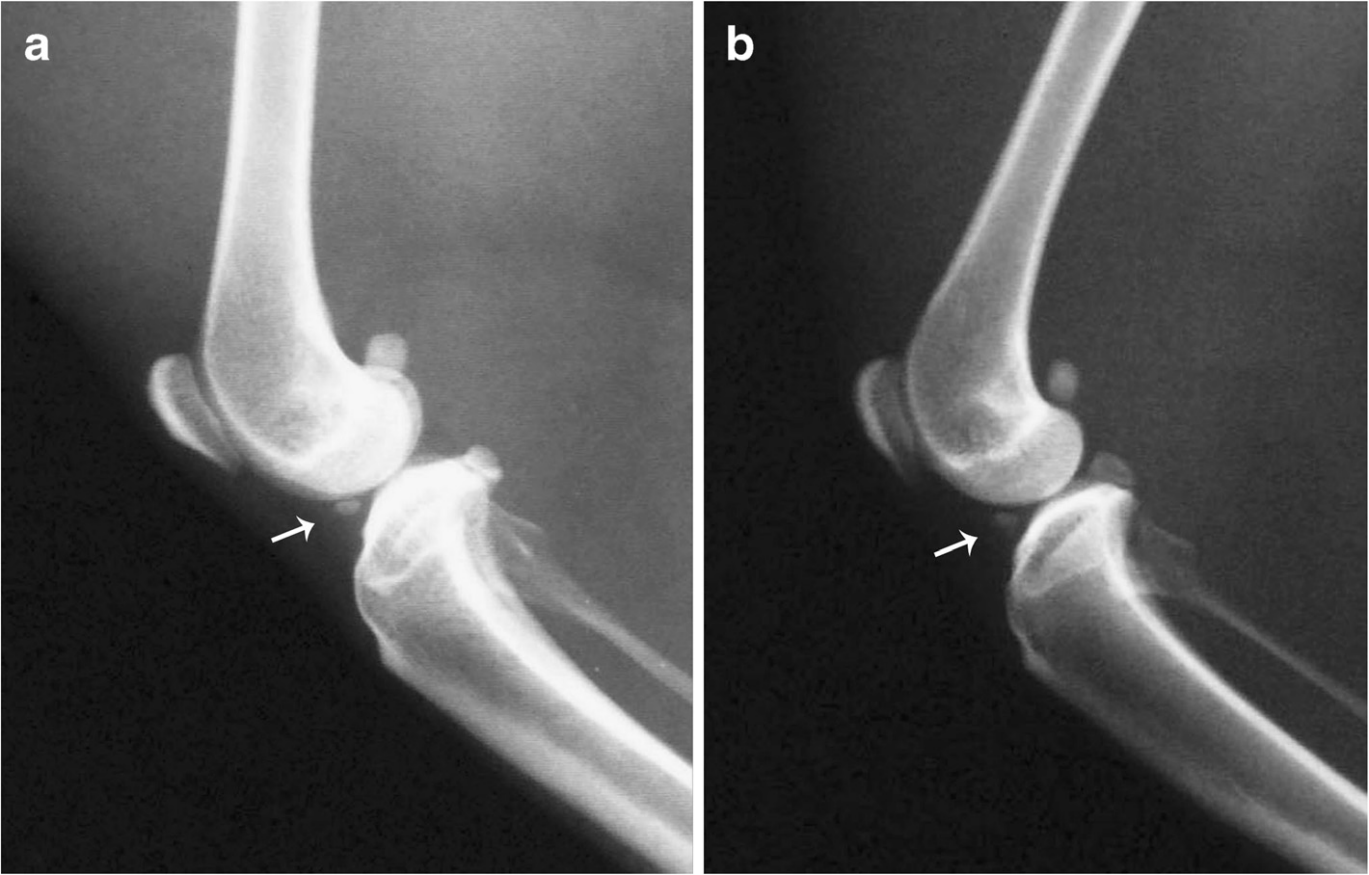
**Mediolateral radiographs of the stifle joints of two little spotted cats.** Observe the meniscal mineralisation (arrow), patella, lateral fabellae and popliteal sesamoid.

**Figure 2:**
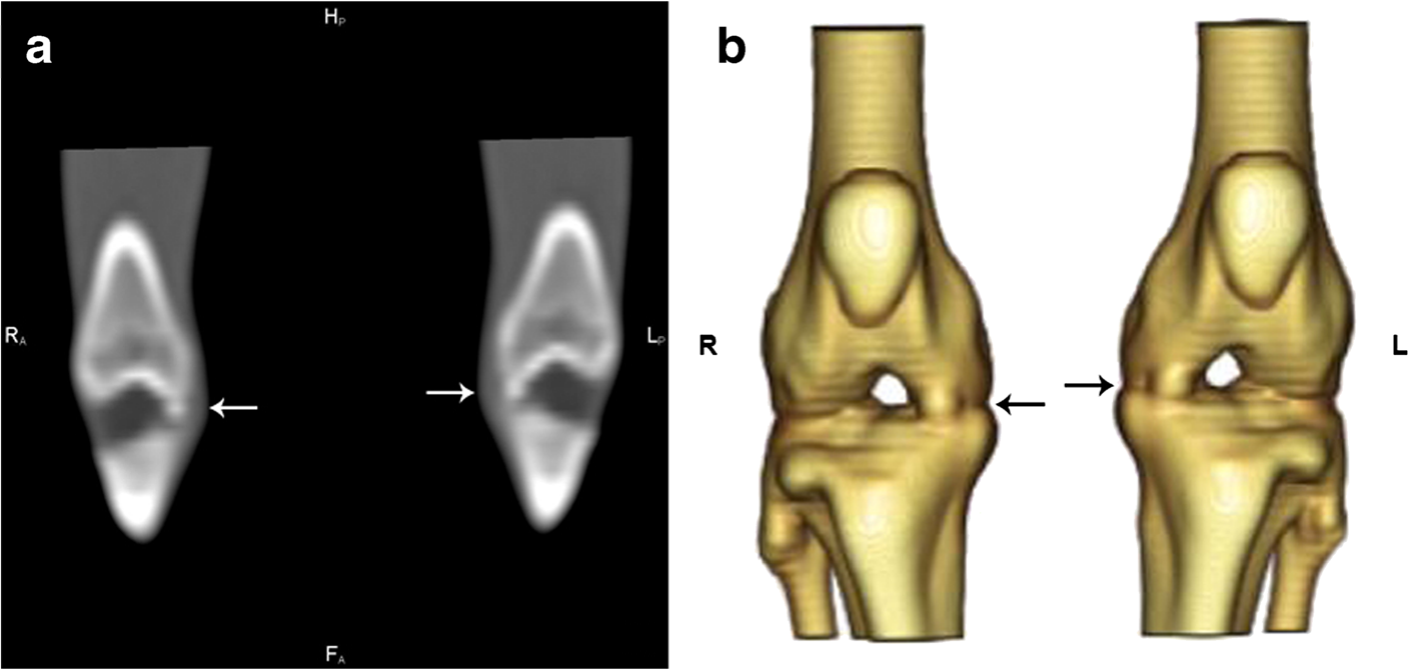
**CT on the coronal plane image and 3D image (cranial view) of the stifle joints of a little spotted cat.** Observe the meniscal mineralisation (arrows).

**Figure 3:**
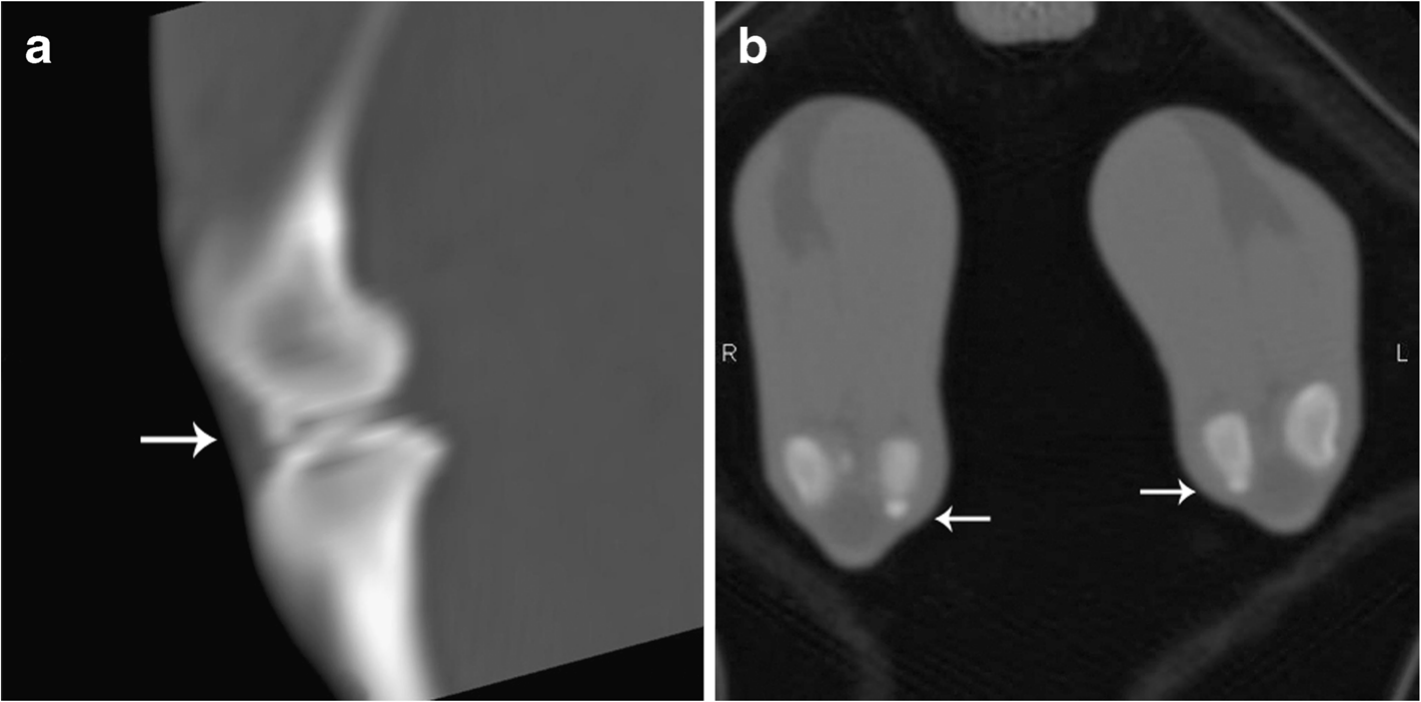
**CT on the sagittal and transverse plane images of the stifle joints of a little spotted cat.** Observe the meniscal mineralisation (arrows).

For one of the animals (n. 1), it was not possible to identify the meniscal mineralisation in either stifle joint on CT or x-ray images (Table 1). The meniscal mineralisation was not visible on CT images in the left stifle of animal n. 6 (Table 1). Radiographically, the meniscal mineralisation was observed as an oval radiopacity in the cranial compartment in all animals on the mediolateral projection, located within the area of the medial meniscus (Figure 1). On craniocaudal projection, the meniscal mineralisation was more difficult to visualise. On CT, meniscal mineralisation was best identified in the transverse plane images (Figures 2 and 3). The meniscal mineralisation was measured at a mean of 0.16 ± 0.04 cm (SD) and at a mean of 0.20 ± 0.07 cm (SD) for the maximum transverse and longitudinal lengths, respectively. The mean medium and maximum HU values were 694.19 ± 206.13 (SD) and 792.14 ± 217.94 (SD), respectively (Table 1).

**Figure 4:**
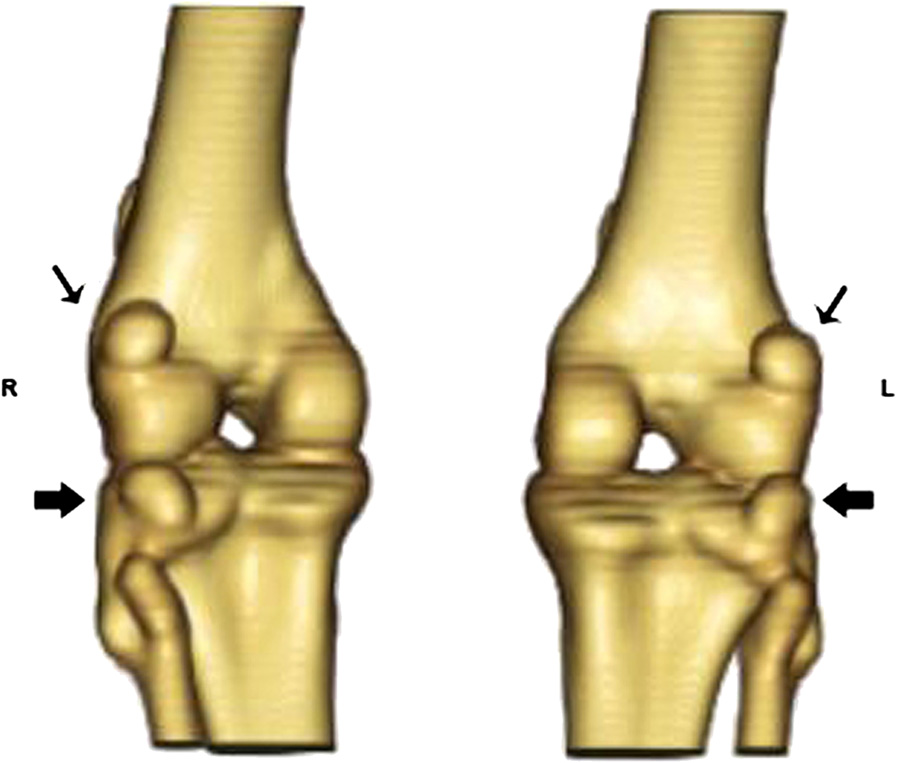
**3D CT image (caudal view) of the stifle joints of a little spotted cat.** Observe the lateral fabellae (narrow arrow) and popliteal sesamoid (thick arrow).

The patella, lateral fabellae and popliteal sesamoid bones were observed in both CT and x-ray images in all animals (Figures 1, 2 and 4). The medial fabellae were not visible. The patella was pear-shaped, with a base and a pointed apex. The lateral fabellae were positioned caudally to the femoral epicondyle on the mediolateral radiographic views and proximally to the lateral femoral condyle on the craniocaudal views. The popliteal sesamoid bone was located at the caudolateral aspect of the stifle joint and was well visualised on the mediolateral radiographs.

**Figure 5:**
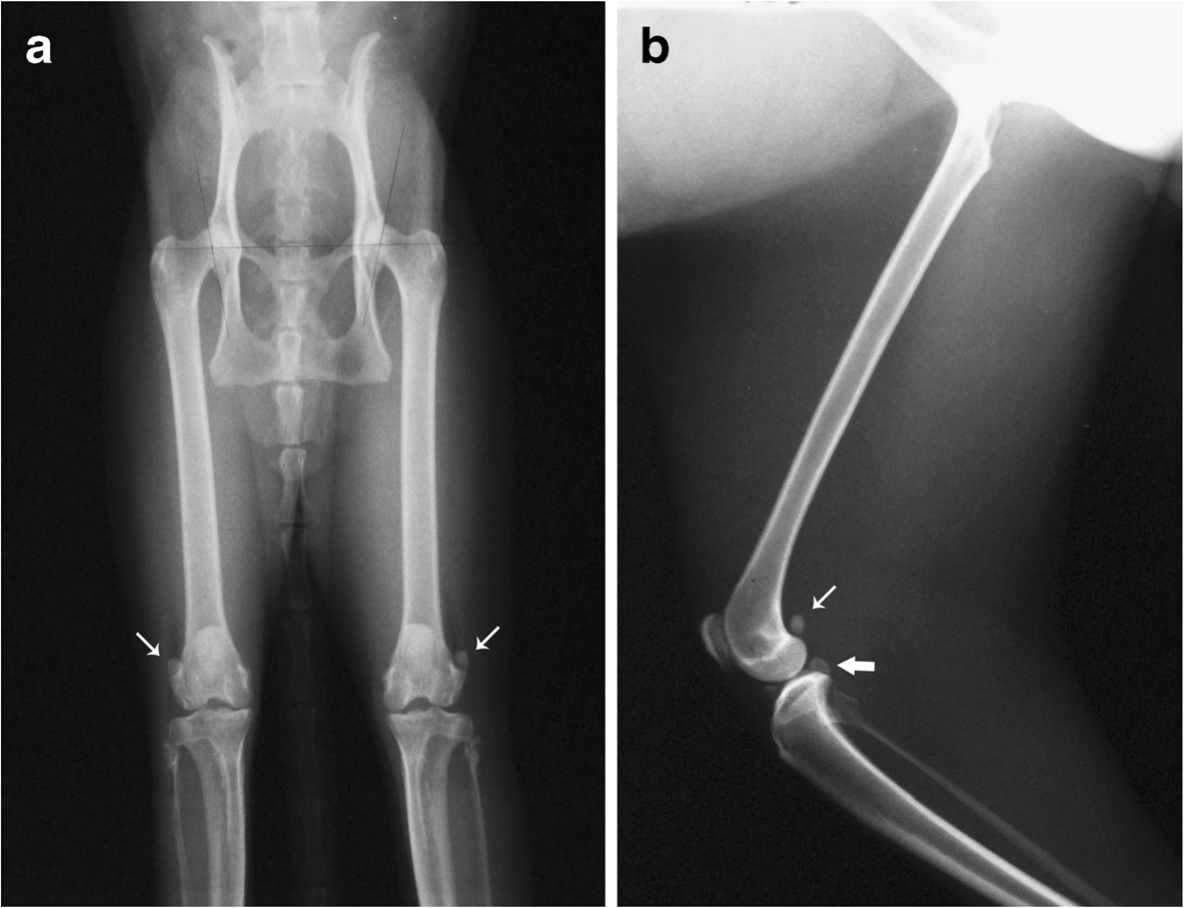
**Ventrodorsal radiographic view of the pelvis and hind limbs (a), and lateral view of the femur (b) of a little spotted cat.** Observe the femoral head, greater trochanter, lateral fabellae (narrow arrow) and popliteal sesamoid (thick arrow). The femoral diaphysis is straight.

Radiographically (Figure 5), the femoral head was hemispherical and connected to the femoral diaphysis by a short neck. The greater trochanter was positioned laterally to the head and neck, and the lesser trochanter was visualised on the caudomedial surface near the junction with the femoral diaphysis. The femoral diaphysis was straight. The femoral condyles were well defined, and the patella fitted into the groove of the trochlea. The proximal extremity of the tibia had lateral and medial condyles and an intercondylar eminence. The head of the fibula articulated with the tibia. The Norberg angle ranged from 100º to 112º (mean ± SD, 106º ± 3.39) (Table 1). There was no evidence of articular disease in either the stifle joints or the hip joints.

## Discussion

All cats had no evidence of joint disease based on imaging. The meniscal mineralization was radiographically visible in 91.67% of the animals in the present study. The aetiology of meniscal mineralisation is not clear [10,11]. There are several different hypotheses for meniscal mineralisation, including primary ossification centres, normal anatomical features of development, and the secondary form, which may be associated with trauma and osteoarthritis [4,9,10,17]. Normal anatomical feature of development seems the hypothesis more likely to apply to meniscal mineralization in non-domestic cats.

In a prospective study [10] using 100 domestic cats, it was observed on radiographs that 45% of them had meniscal mineralisation in one (n = 19) or both (n = 27) stifles. Feline cadavers were also studied, showing that 34 of 57 stifles had meniscal mineralisation located in the cranial horn of the medial meniscus, suggesting medial compartment joint disease. According to the authors, it was not possible to determine whether the meniscal mineralisation was responsible for the cartilage damage or whether it was the result of the lesion. However, although the meniscal mineralisation in the little spotted cat was located in the area of the medial meniscus, no signs of articular degeneration were observed on radiographs and CT images. These findings were similar to those reported in large non-domestic cats, in which the meniscal ossicles are considered to be common and have no correlation with a degenerative process [11,12].

It was also noted that in large non-domestic cats, the ossicles become visible radiographically at approximately one year of age or by the last half of skeletal maturation [11]. However, the only little spotted cat in which meniscal mineralisation was not observed was sufficiently mature to allow this evaluation. Moreover, the HU values of the meniscal mineralisation in 22 joints of the little spotted cat were compatible with bone density [18]. Histological evaluation would be important to associate the values of HU with presence of cancellous or compact bone. In a Bengal tiger, it was observed histologically that the ossicles were composed of dense lamellar bone and did not contain bone marrow [11].

The patella, lateral fabellae and popliteal sesamoid bones are the sesamoid bones that are usually visible on radiographs in the vicinity of the cat’s stifle [19,20]. All of these sesamoids were identified in the present study, and their positions were similar to those in domestic cats [19]. The medial fabellae may be observed in macroscopic and histological studies, but they are often radiolucent on radiographs, especially in pedigree and exotic cats [20]. The medial fabellae were not visible radiographically or on CT images in any of the little spotted cats. However, no animal was euthanised to prove the presence of the medial fabellae. A study of 34 large non-domestic cats reported that some species present a tendency to have a medial meniscal ossicle in concurrence with a lateral fabella, but the reason for this phenomenon was not determined [11]. Although in the present study the lateral fabellae was observed in both CT and x-ray images in all animals, meniscal ossicle was not visible in one little spotted cat.

The radiographic anatomy of the femur in the little spotted cats was very similar to that of healthy domestic cats [19], including having relatively straight bones, with the greater trochanter and the lesser trochanter well visualised. The radiographic signs of hip dysplasia in domestic cats have been associated with a shallow acetabulum, with remodelling and proliferation involving the craniodorsal acetabular margin [21]. In the present study, the femoral head was hemispherical, and no sign of articular disease was detected. In addition, the Norberg angles of the little spotted cat ranged from 101° to 112°. In normal cats, the mean Norberg angle has been reported to be 99.2° and less than that in normal dogs (105º or greater), indicating a shallower acetabulum [22-24].

In a study of 100 domestic cats, osteoarthritis was more prevalent in the elbow, hip, shoulder and tarsal joints and was associated with both increased age and behavioural changes [25]. Although in the present study no signs of articular disease were observed in either the hip or stifle joints, only a small number of animals were studied. On the other hand, most of the animals may be considered to be young, because the maximum longevity in little spotted cats has been reported to be 20 years [13,15]. Thus, more studies using a larger number of older animals will be necessary. One of the major limitations of the present study was that these cats may have had degenerative joint disease, but the radiographs and CT may have not been sensitive enough to recognize early changes. A synovial joint fluid analysis could have helped.

## Conclusions

Meniscal mineralisation appears to be a normal anatomic feature in little spotted cats.

## Competing interests

The authors have declared that no competing interests exist.

## Authors' contributions

SCR and MGF conceived and designed the study; MJM and HSO helped collect the imaging data; CRT and RHT assisted in the data collection methods and FOBM helped draft the manuscript. All of the authors read, contributed to and approved the final manuscript.
